# Plant Protein Disorder: Spatial Regulation, Broad Specificity, Switch of Signaling and Physiological Status

**DOI:** 10.3389/fpls.2022.904446

**Published:** 2022-05-24

**Authors:** An-Shan Hsiao

**Affiliations:** Biosciences, College of Life and Environmental Sciences, University of Exeter, Exeter, United Kingdom

**Keywords:** intrinsically disordered proteins (IDPs), intrinsically disordered region (IDR), liquid–liquid phase separation, spatial regulation of signaling, conformation, protein disorder

## Introduction

Intrinsically disordered proteins (IDPs) are a group of functional proteins without defined 3D structures. Some structured proteins contain ordered domains and functional intrinsically disordered regions (IDRs). Rather than having a single fixed structure, IDPs/IDRs may adopt various conformations depending on different situations (Kim and Han, 2018; Uversky, 2019). Because of the structural flexibility, IDPs/IDRs are not restricted to lock-key modules but rather interact with different partners under different circumstances. Thus, IDPs/IDRs have versatile roles and multiple functions in numerous biological processes (Tompa et al., 2015; Uversky, 2019).

**Figure 1:**
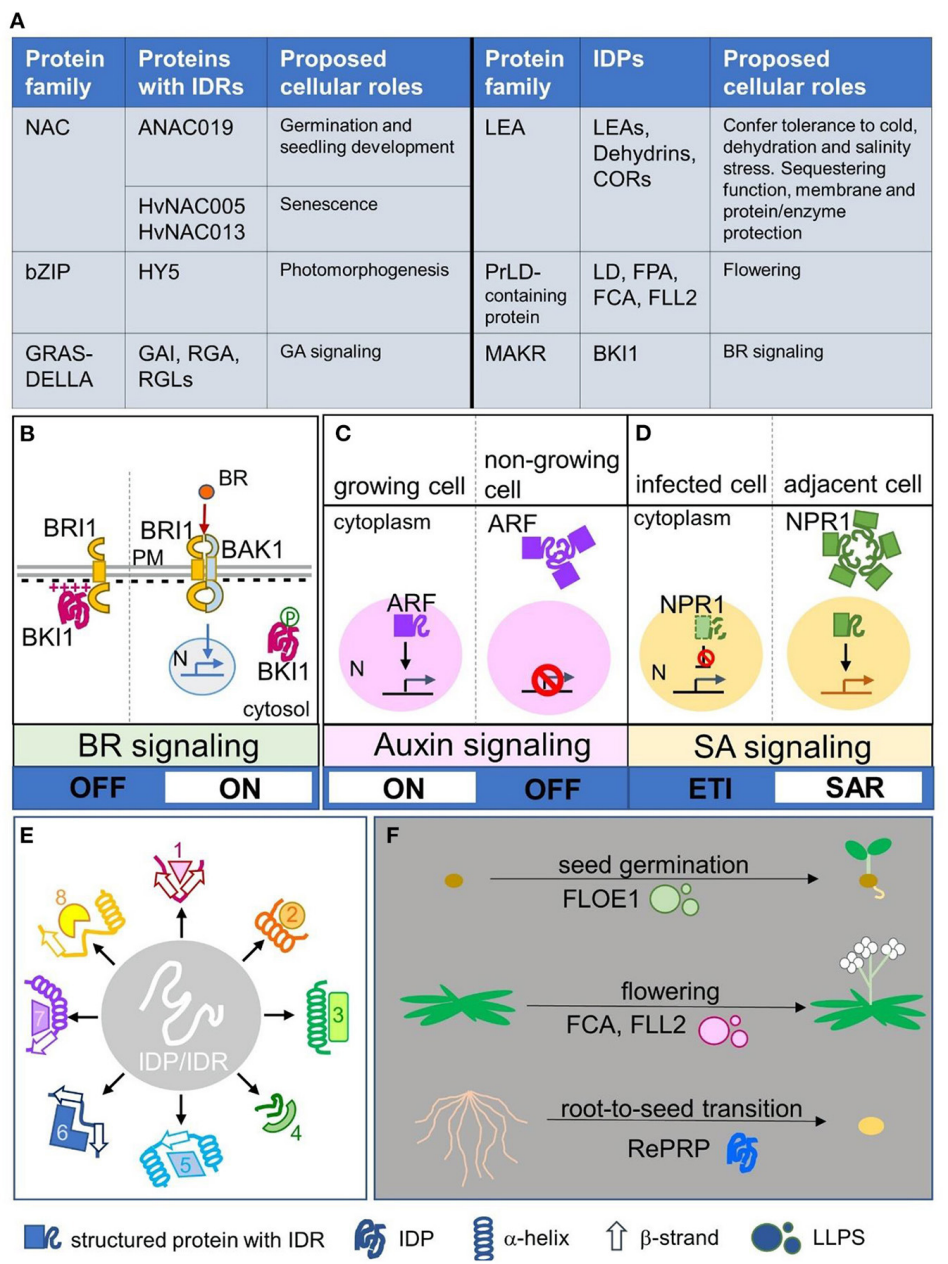
Plant IDP/IDR members and their spatial regulation, broad specificity, and switch of signaling and physiological status. **(A)** Typical examples of plant protein families with IDP members and proteins with IDRs, including their proposed cellular roles. **(B)** A case of spatial regulation of IDPs modulated by electrostatic interaction and post-translational modification. Intrinsically disordered BKI1 associated with the plasma membrane by its disordered cationic region and inhibited the formation of BR receptors (BRI1 and BAK1). Phosphorylation of this region causes electrostatic repulsion to release BKI1 from the plasma membrane into the cytosol; thus BRI1 and BAK1 can form active BR receptors and promote downstream BR signaling. **(C,D)** Two cases showing spatial regulation of IDRs *via* forming biomolecular condensates. **(C)** The nuclear–cytoplasmic compartmentation of ARF mediates auxin signaling turning on or off. In actively growing cells, ARF was present in the nucleus and auxin signaling was activated, whereas in non-growing cells, ARF formed condensates *via* its IDRs and was retained in the cytoplasm; thus auxin signaling was inactivated. **(D)** The nuclear–cytoplasmic compartmentation of NPR1 mediates SA signaling to trigger the ETI or SAR program. In infected cells, the ETI program is promoted to activate programmed cell death, whereas the inhibitor NPR1 is degraded in the nucleus. In adjacent cells, nucleus-localized NPR1 triggers the SAR program, whereas cytoplasmic NPR1 forms biomolecular condensates to inhibit proteins involved in programmed cell death. **(E)** IDPs/IDRs may adopt various secondary conformations such as α-helixes or β-strands when interacting with different partners, which generate broad specificity. **(F)** Three cases showing that protein phase transition controls physiological phase transition. Disordered FLOE1 senses water content and controls seed germination. Disordered FCA and FLL2 form LLPS to control flowering, a phase transition from the vegetative phase to reproductive phase. Intrinsically disordered rice RePRP enables root-to-seed transition as an adaptation response to water deficit stress. IDPs/IDRs: intrinsically disordered proteins/regions; NAC: NO APICAL MERISTEM, ATAF, CUP-SHAPED COTYLEDON; bZIP: basic domain/leucine zippers; HY5: LONG HYPOCOTYL 5; GRAS: GIBBERELLIC ACID INSENSITIVE, REPRESSOR of GAI, SCARECROW; DELLA: Asp-Glu-Leu–Leu-Ala; GAI: GIBBERELLIC ACID INSENSITIVE; RGA: REPRESSOR of GAI; RGL: RGA-LIKE; GA: Gibberellic acid; LEA: LATE EMBRYOGENESIS ABUNDANT; COR: COLD-REGULATED; PrLD: Prion-like domain; LD: Luminidependens; FPA: FLOWERING LOCUS PA; FCA: FLOWERING LOCUS CA; FLL2: FLX-LIKE 2; MAKR: MEMBRANE-ASSOCIATED KINASE REGULATOR; BKI1: BRI1 KINASE INHIBITOR1; BR: brassinosteroid; BRI1: BRASSINOSTEROID INSENSITIVE 1; BAK1: BRI1-ASSOCIATED RECEPTOR KINASE 1; N: nucleus; PM: plasma membrane; ARF: AUXIN RESPONSE FACTOR; NPR1: NONEXPRESSOR OF PATHOGENESIS-RELATED GENE 1; SA: salicylic acid; SAR: systemic acquired resistance; ETI: effector triggered immunity; LLPS: liquid–liquid phase separation; RePRP: REPETITIVE PROLINE-RICE PROTEIN; 1-8 in **(E)** represent different putative interacting partners.

IDRs of transcription factors are proposed to provide functional versatility in molecular recognition *via* their binding plasticity, which facilitates transcriptional regulation of structural domains (Sun et al., 2012). IDPs/IDRs are key factors triggering liquid–liquid phase separation/transition (LLPS/LLPT), which forms membrane-less compartments apart from liquid fluid in a cell, also known as biomolecular condensates, thus allowing the spatiotemporal organization of biochemical reactions by concentrating macromolecules locally (Cuevas-Velazquez and Dinneny, 2018; Kim et al., 2021). In plants, IDRs of transcription factors and signal transduction proteins often form flexible interaction networks or receive various signals, such as plant-specific NAC (for NO APICAL MERISTEM, ATAF, CUP-SHAPED COTYLEDON) transcription factors involved in seed germination and seedling establishment and GRAS (for GIBBERELLIC ACID INSENSITIVE, REPRESSOR of GAI, and the SCARECROW) proteins functioning in gibberellic acid signaling, whereas specific classes of IDPs are involved in flowering and abiotic stress responses (Sun et al., 2013; Covarrubias et al., 2017). Typical examples of plant IDRs/IDPs are shown in Figure 1A. Readers are invited to visit the previous review papers regarding specific topics such as plant IDPs (Sun et al., 2013; Covarrubias et al., 2017), LLPS in plants (Cuevas-Velazquez and Dinneny, 2018; Kim et al., 2021), and dehydrins in stress responses (Cuevas-Velazquez et al., 2014; Graether and Boddington, 2014; Kosová et al., 2014; Yu et al., 2018).

Instead of summarizing the versatile functions of IDPs/IDRs in detail as in the aforementioned review papers, this article highlights the recent breakthroughs in plant IDP/IDR research to provide the whole-picture view; proposes conceptual principles of their action modes on spatial regulation, broad specificity, and signaling/physiological switch; and calls for more research in this emerging field.

## Spatial Regulation Leads to Switch of Signaling

During the late stages of seed maturation, a group of IDPs known as LATE EMBRYOGENESIS ABUNDANT (LEA) proteins are highly expressed in plant seeds before they enter the desiccation phase (Tunnacliffe and Wise, 2007; Leprince et al., 2017), for a strong suggestion of the roles of LEA proteins in desiccation tolerance (Hincha and Thalhammer, 2012). In vegetative tissues, LEA proteins are induced by abiotic stresses such as drought, salinity, heat and freezing (Hincha and Thalhammer, 2012), as noted by the overexpression of LEA proteins often conferring stress tolerance (Samtani et al., 2022). The analysis of LEA proteins in Arabidopsis, tomato and orchid revealed their wide subcellular distribution (Candat et al., 2014; Cao and Li, 2015; Ling et al., 2016), which suggests that their ubiquitous expression would provide protection to the corresponding membranes of various organelles as well as enzymes and sequestering targets localized in different cellular compartments under certain stress conditions (Tunnacliffe and Wise, 2007; Candat et al., 2014; Artur et al., 2019). Dual/multiple subcellular localizations hinting at the spatial flexibility of IDPs/IDRs may be linked to their versatile functions. A wheat IDP, TaFROG, changes its nucleus localization to cytosolic bodies when interacting with SnRK1 (Perochon et al., 2015). Although the mechanism of this spatial transition is still unclear, changing the subcellular localization upon interacting with interaction partners may be a key feature for IDPs/IDRs in regulating downstream signaling.

Intrinsically disordered BRI1 KINASE INHIBITOR1 (BKI1) is a negative regulator of brassinosteroid (BR) signaling (Wang and Chory, 2006). Plasma membrane-localized BKI1 blocked the formation of BR receptor complex, consisting of BRASSINOSTEROID INSENSITIVE 1 (BRI1) and BRI1-ASSOCIATED RECEPTOR KINASE 1 (BAK1), whereas cytosolic BKI1 allows BRI1 and BAK1 to stably interact and initiate downstream BR signaling (Wang and Chory, 2006; Belkhadir and Jaillais, 2015). Figure 1B shows the BKI1 working model. The plasma membrane–cytosol translocation of BKI1 is controlled by the disordered cationic region, which interacts with anionic lipids, and phosphorylation of this region causes electrostatic repulsion to release BKI1 from the plasma membrane into the cytosol (Novikova et al., 2021). Such tunable membrane association regulated by electrostatic interaction and post-translational modification has been reported in other IDPs/IDRs. For example, the binding of intrinsically disordered dehydrin Lti30 to lipid membrane vesicles is regulated by protonation/deprotonation and phosphorylation/dephosphorylation (Eriksson et al., 2011). Hence, electrostatic interaction or post-translational modification seems to be a common mechanism for IDPs/IDRs to regulate the spatial translocation, which might affect the downstream signaling switch.

Recent breakthroughs highlighted that spatial regulation mediated by IDRs *via* condensate formation leads to a switch of hormone signaling regarding development and immune responses. AUXIN RESPONSE FACTORS (ARFs) control genome-wide transcriptional responses to auxin (Lavy and Estelle, 2016). In actively growing tissues, activating ARF proteins are present in the nucleus to turn on the expression of auxin-response genes (Lavy and Estelle, 2016). In tissues that no longer need to respond to auxin, IDRs of ARF drive protein assemblies in the cytoplasm, thus turning off auxin signaling (Powers et al., 2019). Figure 1C shows the ARF working model. ARF nuclear–cytoplasmic partitioning is a mechanism to control auxin responsiveness in specific tissues, whereas the IDR is necessary for cytoplasmic ARF condensate formation to switch off auxin signaling (Powers et al., 2019). In the plant immune response, salicylic acid (SA), through its receptor NONEXPRESSOR OF PATHOGENESIS-RELATED GENE 1 (NPR1), activates effector triggered immunity (ETI) in local tissues and systemic acquired resistance (SAR) in distal tissues (Withers and Dong, 2016). During the ETI response, SA-induced stress response proteins result in rapid localized programmed cell death in infected cells (Coll et al., 2011). In adjacent cells with low pathogen load, SA-mediated activation of nuclear NPR1 promotes SAR gene expression to activate the cell survival program, whereas cytoplasmic condensates formed by the IDR of NPR1 serves as an interacting hub to sequester and degrade proteins involved in programmed cell death (Zavaliev et al., 2020). Figure 1D shows the NPR1 working model. Nuclear–cytoplasmic dual localization of NPR1 is involved in the decision of cell death or survival during ETI and the SAR response, whereas the IDR of NPR1 plays a critical role in switching the programming (Zavaliev et al., 2020).

## Conformational Flexibility Enables Broad Specificity

IDPs/IDRs can undergo disorder-to-order transition and adopt ordered secondary structures such as α-helixes or β-strands upon dehydration and in the presence of their binding partners (Thalhammer et al., 2014; Suarez et al., 2015; Cuevas-Velazquez et al., 2016; Bremer et al., 2017; Saucedo et al., 2017). For example, disordered LEA proteins can acquire folded structures during stress conditions, and this structural plasticity is proposed to be essential for their functions in sequestering and enzyme/membrane protection (Tunnacliffe and Wise, 2007; Cuevas-Velazquez et al., 2016; Artur et al., 2019). The various conformations of IDPs/IDRs under different circumstances generate their broad specificity (Uversky, 2019), as represented in Figure 1E. In the plant defense response, RPM1-INTERACTING PROTEIN 4 (RIN4) is a negative regulator and targeted by multiple bacteria effectors (Belkhadir et al., 2004; Kim et al., 2005; Luo et al., 2009). The IDR of RIN4 folds partly into α-helixes and partly into β-strands to interact with the bacterial effector AvrB (Desveaux et al., 2007), whereas other conformations folded by the IDR such as α-helixes, β-strands, and irregular structures are proposed when RIN4 interacts with other partners (Sun et al., 2014). The latest report suggested that the intrinsically disordered nature of RIN4 provides a flexible platform to broaden pathogen recognition specificity by establishing compatibility with otherwise incompatible leucine-rich repeat immune receptor proteins (Kim et al., 2022). Such platforms formed by IDPs/IDRs play a crucial role in the dynamic host–pathogen interaction (Rikkerink, 2018; Ceulemans et al., 2021). Protein disorder is important in the plant immune system (Marín and Ott, 2014), and disorder in pathogen effectors may also have a versatile contribution to virulence (Marín et al., 2013), supported by a recent report that the disordered AVR2 effector escapes host recognition (Yang et al., 2020). It is a continuing race of disorder between plant hosts and pathogens.

During stress, stress granules are formed *via* LLPS to store mRNA and repress translation (Maruri-López et al., 2021). Highly disordered tudor staphylococcal nuclease (TSN) associating with stress granule proteins *via* its N-terminal IDR acts a docking platform for a protein–protein interaction network to enable rapid stress granule assembly under stress (Gutierrez-Beltran et al., 2021). Small heat shock proteins (sHSPs) protect cells against stress-induced cell damage by binding to and maintaining denaturing proteins in a folding-competent state (Wu et al., 2022). The N-terminal IDR of pea sHsp18.1 presents a variable and flexible ensemble with multiple binding site conformations; hence, sHSPs are highly effective in interacting in a wide range of different cellular proteins (Jaya et al., 2009). Such interacting hubs formed by IDPs/IDRs awaits more direct structural evidence to verify their conformation. Nuclear magnetic resonance (NMR) spectroscopy can probe IDP/IDR interactions and has made significant contributions with the description of the binding mechanisms and the mapping of the conformational dynamics of IDPs/IDRs (Schneider et al., 2019). Although technical challenges remain, in-cell NMR will be a possible solution to observe the conformational changes of IDPs/IDRs upon binding, post-translation modification and in response to environmental stimuli (Cedeño et al., 2017).

## Protein Phase Transition Controls Physiological Phase Transition

Plant development undergoes several distinct phases: germination, vegetative growth, flowering, seed setting and senescence (Bäurle and Dean, 2006). The transitions between these phases are controlled by distinct genetic circuits that integrate endogenous and environmental cues (Bäurle and Dean, 2006; Huijser and Schmid, 2011). Disordered proteins have phase transition properties to undergo LLPS and form biomolecular condensates (Majumdar et al., 2019). Notably, this phenomenon is also involved in plant developmental-phase transition such as flowering and seed germination. Prion-like domains (PrLDs) are intrinsically disordered and identified as drivers for LLPS (Malinovska et al., 2013; Patel et al., 2015). In plants, PrLD-containing proteins are associated with diverse stress and memory processes (Garai et al., 2021), important for the assembly of stress granules (Kosmacz et al., 2019) and functional for flowering transition (Chakrabortee et al., 2016; Huang et al., 2022). The regulation of the Arabidopsis floral repressor *FLC* involves RNA 3′-end processing, which reduces *FLC* transcriptional initiation and elongation to stimulate flowering (Whittaker and Dean, 2017). The PrLDs of FLOWERING LOCUS CA (FCA) undergo LLPS to form condensates, called FCA nuclear bodies (Fang et al., 2019). Highly disordered FLX-LIKE 2 (FLL2) could promote phase separation of FCA nuclear bodies, interact with polymerase and nuclease modules to form a functional RNA 3′-end processing machinery, thus enhancing polyadenylation at specific sites to reduce transcriptional read-through and controlling flowering time (Fang et al., 2019).

A recent study revealed that the dry-to-wet phase transition of Arabidopsis IDP FLOE1 is involved in seed germination (Dorone et al., 2021). FLOE1 is usually dispersed throughout a dry seed but rapidly forms LLPS blobs when exposed to water (Dorone et al., 2021). The phase transition of FLOE1 acts as a water sensor to regulate seed germination and prevents seeds from sprouting in unfavorable conditions (Dorone et al., 2021). Seeds are desiccation-tolerant (Giarola et al., 2017), with a physiological stage similar to anhydrobiotes (Boothby and Pielak, 2017); unsurprisingly, a physiological status mimicking desiccation-tolerant seeds would defeat the water deficit. A root-to-seed transition concept for overcoming water deficit was presented in a recent study of a rice IDP, REPETITIVE PROLINE-RICE PROTEIN (RePRP) (Hsiao et al., 2020). RePRP halted root growth by inhibiting cell elongation *via* interacting with actin and microtubule cytoskeleton, whereas the quiescent root accumulated more nutrients (i.e., starch) under water deficit, thus shifting the roots to a dormant storage organ resembling seeds (Hsiao et al., 2020). Figure 1F shows the aforementioned examples of physiological phase transition. Manipulating the protein phase transition of IDPs/IDRs may be a good tool to control plant physiological stages. A recent study analyzed 96 plant proteomes and revealed that the Poaceae family has the most abundant IDPs/IDRs as compared with land plant clades (Zamora-Briseño et al., 2021). The abiotic stress-tolerant bioenergy crop switchgrass and desiccation-tolerant resurrection grass had the highest proportion of proteins with intense disorder (Zamora-Briseño et al., 2021), which suggests that IDPs/IDRs may play critical roles in plant stress responses and serve as potential engineering targets for climate-resilient crop plants.

## Conclusions

The plasticity of IDPs/IDRs may be an easy and fast way for sessile plants to introduce versatility into protein interaction networks to quickly and efficiently adapt to environmental changes (Pietrosemoli et al., 2013). For potential application of plant IDPs/IDRs in agriculture, we need to investigate their spatial regulation and conformation–function relation in signaling and the physiological status. This emerging field merits more research attention.

## Author Contributions

A-SH developed the concept and wrote the manuscript. The author contributed to the article and approved the submitted version.

## Funding

A-SH was supported by a BBSRC research grant (BBSRC BB/T005424/1) led by Prof. Nicholas Smirnoff. This study did not generate any new data.

## Conflict of Interest

The author declares that the research was conducted in the absence of any commercial or financial relationships that could be construed as a potential conflict of interest.

## Publisher's Note

All claims expressed in this article are solely those of the authors and do not necessarily represent those of their affiliated organizations, or those of the publisher, the editors and the reviewers. Any product that may be evaluated in this article, or claim that may be made by its manufacturer, is not guaranteed or endorsed by the publisher.
